# Supplementary material for: The causal role of circulating inflammatory markers in osteoporosis: a bidirectional Mendelian randomized study
Source: Front Immunol. 2024 Jul 18;15:1412298. doi: 10.3389/fimmu.2024.1412298 (PMC11291241; doi:10.3389/fimmu.2024.1412298)
Supplement: Supplementary file 1 [file DataSheet_1.zip › supplementary material/OSTEOPOROSIS-CIM/GCST90274776.tsv.gz_leave_one_out.pdf]

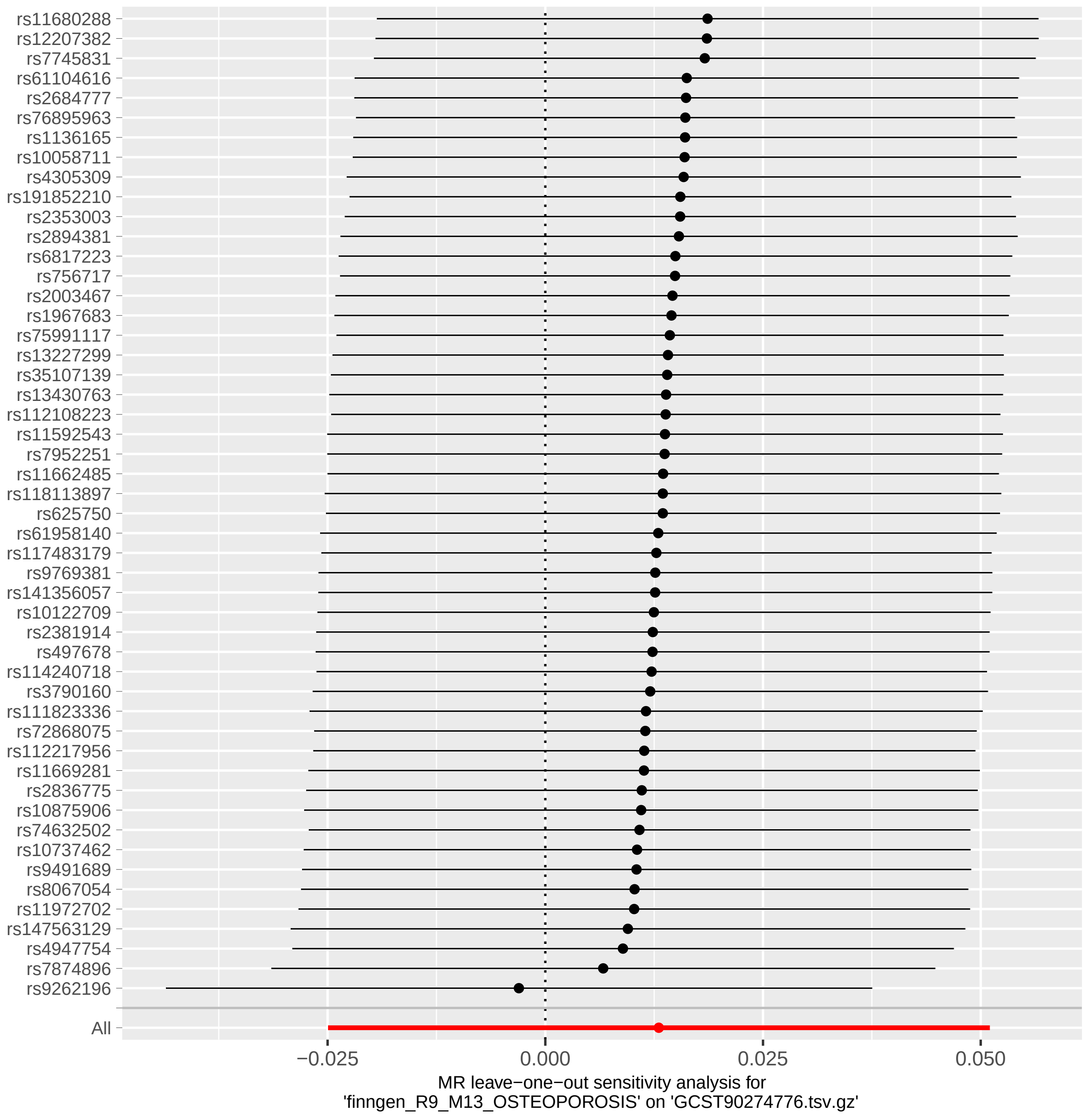
rs11680288
rs12207382
rs7745831
rs61104616
rs2684777
rs76895963
rs1136165
rs10058711
rs4305309
rs191852210
rs2353003
rs2894381
rs6817223
rs756717
rs2003467
rs1967683
rs75991117
rs13227299
rs35107139
rs13430763
rs112108223
rs11592543
rs7952251
rs11662485
rs118113897
rs625750
rs61958140
rs117483179
rs9769381
rs141356057
rs10122709
rs2381914
rs497678
rs114240718
rs3790160
rs111823336
rs72868075
rs112217956
rs11669281
rs2836775
rs10875906
rs74632502
rs10737462
rs9491689
rs8067054
rs11972702
rs147563129
rs4947754
rs7874896
rs9262196
All
−0.025
0.000
0.025
0.050
MR leave−one−out sensitivity analysis for
'finngen_R9_M13_OSTEOPOROSIS' on 'GCST90274776.tsv.gz'
